# The associative pathways linking intangible cultural heritage short videos to youth rural cultural identity: An experiment based on eye-tracking and structural equation modeling

**DOI:** 10.3389/fpsyg.2026.1831646

**Published:** 2026-06-25

**Authors:** Haozhuo Lin, Qiu Chen

**Affiliations:** 1 School of Marxism, Wenzhou University of Technology, Wenzhou, Zhejang, China; 2 School of Humanities, Wenzhou University, Wenzhou, Zhejang, China

**Keywords:** eye-tracking, intangible cultural heritage short videos, mediating effects, rural cultural identity, structural equation modeling

## Abstract

Against the background of digital media development and rural cultural revitalization, intangible cultural heritage (ICH) faces intergenerational inheritance challenges among young people. Existing relevant studies mainly adopt subjective analysis and lack objective quantitative exploration of its influencing mechanisms. This study combined eye-tracking experiments and questionnaire surveys, collecting 62 matched datasets and 418 valid questionnaires for empirical analysis. The results revealed that ICH short video characteristics positively affected youth's visual attention, which further triggered emotional resonance and cultural belonging; rural contact frequency played a positive moderating role in this process. Visual attention, emotional resonance and cultural belonging formed significant parallel and chain mediating effects between ICH short videos and rural cultural identity. This research clarifies the internal associative pathways and provides practical references for the digital communication of ICH and the cultivation of youth rural cultural identity.

## 1 Introduction

Intangible cultural heritage constitutes the core of China's outstanding traditional culture, embodying the spiritual essence of agrarian civilization. It preserves the nation's historical memory, value systems, and cultural DNA, serving as a living medium for rural cultural transmission (Shakya and Vagnarelli, 2024). Amid rapid urbanization and digital cultural evolution, the physical and emotional bonds between youth and rural communities are weakening, resulting in a severe intergenerational gap in rural cultural inheritance (Ibrahim and Yuningsih, 2025). Short videos, characterized by their lightweight, visual, social, and low-threshold nature, have become the primary medium for young people (Okopnyi et al., 2023). By breaking down barriers of time, space, and expertise, these videos transform niche intangible cultural heritage (ICH) skills and folk rituals into interactive digital content. Platforms like Douyin's “ICH Inheritors” accounts and Kuaishou's “Local ICH” topics (with over 50 billion cumulative views) have become key channels for young people to explore local culture (Giovannini et al., 2021).

The dissemination of short videos on ICH still faces significant challenges. On one hand, some creators excessively pursue traffic by entertaining and sensationalizing ICH adaptations, resulting in superficial understanding of rural culture among young people (Dezfoulian and Nemati, 2025). On the other hand, existing research predominantly focuses on describing communication effects, lacking in-depth exploration of the internal mechanisms through which short videos relate to youth's rural cultural identity. Moreover, the absence of objective empirical data undermines the scientific basis for developing effective communication strategies (Lou et al., 2025). From the perspective of cognitive processing, the association of short videos with audiences begins with visual attention. The allocation and intensity of attention closely relate to the depth of processing for intangible cultural heritage information, thereby influencing the formation of emotional resonance and cultural identity (Sheets et al., 2023). Traditional questionnaires rely on subjective reports, which can introduce bias. In contrast, eye-tracking technology objectively records key metrics such as total fixation time and the proportion of fixation in interest areas, reflecting authentic cognitive states (Frautschi et al., 2020). Structural equation modeling (SEM) can handle complex multivariate relationships and test direct, indirect, and moderating effects (Almeida, 2024).

Based on this, this study integrates eye-tracking experiments and structural equation modeling to systematically explore the internal associative pathways linking intangible cultural heritage short videos with youth rural cultural identity, providing empirical evidence consistent with the digital inheritance and youth cultural identity cultivation pathway.

## 2 Theoretical basis

### 2.1 Stimulus-Organism-Response theory (S-O-R)

The S-O-R theory posits that environmental external stimuli (S) relate to an organism's internal psychological state (O), which then triggers behavioral responses (R), with the internal state serving as the mediator between stimuli and reactions (Dashti et al., 2019). This study applies the theory to the dissemination of intangible cultural heritage (ICH) short videos: external stimuli (S) include visual salience, narrative completeness, and interactive immersion; internal psychological states (O) encompass young people's visual attention, emotional resonance, and cultural belonging (Fadlah et al., 2025); behavioral and psychological responses (R) involve their identification with local culture (including cognitive, emotional, value, and behavioral recognition; Granic et al., 2020). This framework provides a core structure for clarifying the logical relationships among variables and formulating research hypotheses.

### 2.2 Theory of limited attention

The limited attention theory posits that individuals' attention resources are finite, leading them to selectively focus on information that is both appealing and relevant to their needs. The visual salience and content structure of information serve as key external factors influencing attention allocation (Amin et al., 2021). In this study, young people face a deluge of short video content. Only those with high visual salience, coherent narratives, and strong interactivity can capture their visual attention and prompt deeper cognitive processing. This theory supports the hypothesized association between short video characteristics and visual attention (Mesana et al., 2024).

### 2.3 Social identity theory

Social identity theory posits that individuals develop group identity through social categorization, comparison, and identification, which in turn fosters a sense of belonging and pride. Group culture and symbols serve as crucial foundations for social identity formation (Charness and Chen, 2020). Rural cultural identity represents the cultural manifestation of social identity. By watching short videos on intangible cultural heritage, young people gain insights into rural cultural connotations, integrate into rural cultural groups, and cultivate cultural belonging and rural cultural identity (Cao, 2025). This theory underpins the hypothesized sequential associations among emotional resonance, cultural belonging, and rural cultural identity.

### 2.4 Theories of affective-cognitive evaluation

The affective cognitive evaluation theory posits that emotions arise from an individual's cognitive assessment of external stimuli, with cognitive evaluation serving as the prerequisite for emotional generation. Positive emotional experiences foster the development of positive attitudes and behaviors (Doan et al., 2025). In this study, young participants engaged in cognitive processing of ICH short videos through visual attention. When they perceived the ICH culture as aligning with their personal needs and values, it triggered positive emotional resonance such as emotional impact and pride, thereby strengthening their cultural belonging and identification with local traditions (Edson, 2004). This theory provides empirical support for the hypothesized sequential associative relationships among visual attention, emotional resonance, and local cultural identity.

## 3 Research hypotheses and model construction

### 3.1 Research hypotheses

Based on the literature review and theoretical foundation, and following the theoretical associative framework of short video characteristics, visual attention, mediating variables, and rural cultural identity, 12 research hypotheses covering direct, mediating, and moderating effects are proposed.

Based on the limited attention theory, the following hypotheses are proposed: H1: The visual salience of ICH short videos is significantly positively associated with young people's visual attention; H2: The narrative completeness of ICH short videos is significantly positively associated with young people's visual attention; H3: The interactive immersion of ICH short videos is significantly positively associated with young people's visual attention.

According to the theory of affective cognitive evaluation, the following hypotheses are put forward: H4: Visual attention is significantly positively associated with the emotional resonance of young people; H5: Visual attention is significantly positively associated with the cultural belonging of young people.

According to the social identity and emotional cognition evaluation theory, the following conclusions are drawn: H6: Emotional resonance is significantly positively associated with the young people's identification with the local culture; H7: cultural belonging is significantly positively associated with the young people's identification with the local culture.

Based on the S-O-R theory, the characteristics of ICH short videos indirectly relate to rural cultural identity through internal psychological states. The study proposes the following hypotheses: H8: Visual attention plays a mediating role in the association between ICH short video features and youth rural cultural identity; H9: Emotional resonance plays a mediating role in the association between visual attention and youth rural cultural identity; H10: Cultural belonging plays a mediating role in the association between visual attention and youth rural cultural identity; H11: The sequential mediating role of visual attention, emotional resonance, and cultural belonging is supported between ICH short video features and youth rural cultural identity.

The rural contact frequency reflects the degree of connection between youth and rural culture. The higher the contact frequency, the easier it is to transform visual attention into emotional resonance. It is proposed that H12: The rural contact frequency positively moderates the association between visual attention on emotional resonance, meaning the higher the rural contact frequency, the stronger the positive association of visual attention on emotional resonance.

### 3.2 Theoretical model construction

Based on the aforementioned research hypotheses, this study constructs a theoretical model examining associative pathways linking ICH short videos with youth rural cultural identity. The model clarifies the logical relationships among variables as follows: (1) Independent variables: Three dimensions of ICH short videos—visual salience, narrative completeness, and interactive immersion—serve as external stimuli associated with youth's visual attention and rural cultural identity. (2) Mediating variables: Visual attention (cognitive processing mediator), emotional resonance (emotional experience mediator), and cultural belonging (affiliation reinforcement mediator) collectively form the mediating chain connecting independent and dependent variables. (3) Dependent variable: Rural cultural identity, comprising four dimensions—cognitive, emotional, value, and behavioral identification—represents the ultimate psychological response triggered by ICH short videos through mediating variables. (4) Moderating variable: Rural exposure frequency, which regulates the relationship between visual attention and emotional resonance. (5) Control variables: Gender, age, education level, major (whether in cultural-related fields), and daily short video usage duration are selected to control for interference with the dependent variable, ensuring the accuracy of research findings.

The core logic of the theoretical model in this study is that the visual salience, narrative completeness, and interactive immersion of short videos on intangible cultural heritage (ICH) are associated with the visual attention of young people, which then relates to their identification with rural culture through parallel and chain mediations of emotional resonance and cultural belonging. Meanwhile, the frequency of rural exposure moderates the transformation process from visual attention to emotional resonance.

To quantify the comprehensive level of visual attention, this study developed a calculation formula for the Visual Attention Synthesis Index (VA). This index integrates four core eye-tracking metrics—total fixation duration, average fixation duration, fixation count, and AOI fixation proportion—into a single indicator, based on established theoretical and methodological grounds in cognitive psychology and communication eye-tracking research.

Total fixation duration reflects the overall attentional investment and sustained engagement; average fixation duration indicates the depth of cognitive processing and elaboration on content; fixation count represents the frequency and distribution of attentional focus; and AOI fixation proportion measures the selectivity and concentration of attention on core ICH information. These four metrics capture complementary dimensions of visual attention: overall engagement, processing depth, attention frequency, and target selectivity. Collectively, they form a comprehensive and theoretically coherent characterization of visual attention without conceptual overlap, justifying their aggregation into a single synthesis index.

Before aggregation, we conducted dimensionality and reliability tests to validate the unidimensionality and internal consistency of the four eye-tracking metrics. Exploratory factor analysis (EFA) was performed on the 738 valid eye-tracking data points. The results showed a single dominant factor with an eigenvalue of 2.87, explaining 71.75% of the total variance, supporting unidimensionality. Internal consistency was assessed using Cronbach's α, which reached 0.82, well above the 0.70 threshold, indicating high reliability among the four metrics. These tests confirm that the four indicators belong to a single latent construct of visual attention and are suitable for integration into a composite index.

Areas of interest (AOIs) were defined consistently and uniformly across all 12 stimulus videos to ensure comparability. AOIs were manually delineated in Tobii Pro Lab to cover core ICH content, including: Demonstration areas of ICH crafts and skills, Faces and operating hands of ICH inheritors, Scenes of rural cultural rituals and folk activities, Text and labels explaining ICH connotations. AOIs were defined using the same semantic and spatial rules for all 12 videos. AOI size was normalized by the total screen area to eliminate differences caused by video composition. All AOIs were verified by two independent coders (inter-coder reliability = 0.91) to ensure consistency. This standardization ensures that AOI fixation proportion is comparable across all stimulus materials and accurately reflects attention to core ICH content.

The formula (1) is displayed below:


$$\begin{array}{l} VA=\frac{1}{4}\left(Z_{TFD}+Z_{AFD}+Z_{AFR}\right) \end{array}$$ (1)

Here, *VA* represents the Visual Attention Synthesis Index, with Z_*TFD*_ being the standardized total fixation duration, Z_*AFD*_ the average fixation duration, Z_*FD*_ the fixation frequency, and Z_*AFR*_ the fixation proportion in the region of interest. *Z*-score normalization eliminates dimensional differences among metrics, and the mean value is taken as the synthesis index to achieve a single-dimensional quantitative representation of visual attention.

To assess the total effect value of chain-mediated effects, this study employed the chain-mediated total effect calculation formula, integrating the product relationships of path coefficients. Formula (2) is displayed below:


$$\begin{array}{l} TE_{chain}=a\times b\times c \end{array}$$ (2)

Here, TE_*chain*_ represents the total chained mediating effect among the sequential associations of visual attention, emotional resonance, and cultural belonging. *a* denotes the path coefficient of intangible cultural heritage short video features on visual attention, *b* represents the path coefficient of visual attention on emotional resonance, and *c* indicates the path coefficient of emotional resonance on cultural belonging. The chain-mediated effect is quantitatively calculated through the multiplicative product of these path coefficients.

To examine the moderating effect of rural contact frequency, this study developed a regression model incorporating interaction terms for quantitative analysis. The formula (3) is displayed below:


$$\begin{array}{l} EC=\beta_{0}+\beta_{1}\times VA+\beta_{2}\times FHC+\beta_{3}\times \left(VA\times FHC\right)\\ \;+\Sigma \beta_{1}\times CV_{i}+\varepsilon \end{array}$$ (3)

Here, *EC* represents emotional resonance, *VA* denotes visual attention, *FHC* stands for rural contact frequency, and *VA*×*FHC* is the interaction term between visual attention and rural contact frequency. CV_*i*_ is the control variable, β_0_ is the intercept term, β_1_,β_2_, β_3_, and β_*i*_ are the path coefficients of each variable, and ε is the random error term. A significant β_3_ indicates the moderating effect of rural contact frequency.

**Figure 1:**
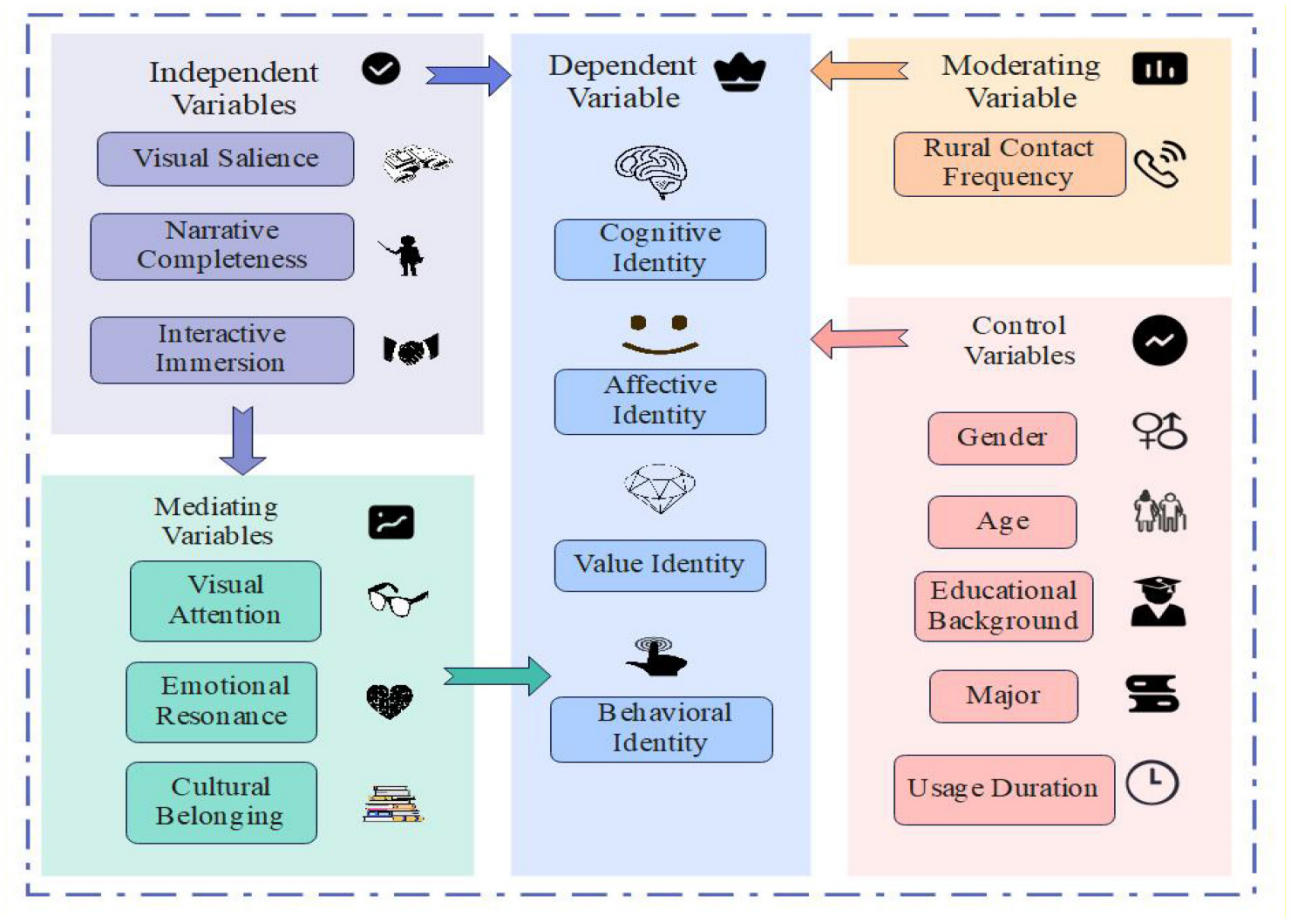
Theoretical model diagram.

The diagram of the theoretical model is shown in Figure 1.

## 4 Study design

This study adopted a mixed research design of “experimental study + quantitative questionnaire,” combined with eye-tracking experiment and questionnaire survey to obtain objective visual attention data and subjective attitude data, and tested the research hypothesis through statistical analysis to reveal the associative pathways mechanism, and strictly followed the scientific research norms to ensure the rationality of the design, the objectivity of the data and the rigor of the analysis.

### 4.1 Mixed study design approach

Eye-tracking experiment: Using an eye tracker to record young participants' eye movement data while watching short videos of intangible cultural heritage, we obtained objective visual attention metrics such as total fixation duration and fixation proportion in interest areas, thereby avoiding subjective reporting bias. Questionnaire survey: Post-experiment, participants provided subjective data including evaluations of video features, emotional resonance, and cultural belonging to comprehensively assess their psychological state. Data integration and analysis: By combining eye-tracking and questionnaire data, we conducted reliability and validity analysis, structural equation modeling, and Bootstrap mediation effect testing to validate the research hypotheses.

### 4.2 Eye-tracking experiment design

The Tobii Pro Fusion desktop eye tracker, with a sampling rate of 120Hz, can record eye movement trajectories in real time (Orduna-Hospital et al., 2023). It features a 27-inch display with a resolution of 1920 × 1080 to ensure video clarity, and a tracking range of 30–120 cm, making it suitable for laboratory settings (Henry et al., 2022). Paired with the Tobii Pro Lab software, it enables precise extraction and analysis of core eye movement metrics (Zhang and Cui, 2022).

The study subjects were young adults aged 18–35 years with normal visual acuity (or corrected normal vision), a habit of watching short videos, and basic knowledge of intangible cultural heritage. A total of 68 participants were recruited through a combination of online and offline methods. Six participants were excluded due to low eye-tracking rates (below 70%) or early withdrawal, resulting in 62 valid participants with an effective rate of 91.18%. Demographic characteristics are shown in Table 1.

**Table 1:** Demographic characteristics of valid participants in the eye-tracking experiment (*N* = 62).

Demographic characteristics	Class	Number of people	Proportion (%)
Sex	The male sex	19	30.6
	Femininity	43	69.4
Age	18–22 years old	28	45.2
	23–28 years old	21	33.9
	29–35 years old	13	21.0
Record of formal schooling	Undergraduate or lower	42	67.7
	Master's degree or above	20	32.3
Specialty	Culture-related	27	43.5
	Non-culture-related	35	56.5
Average daily short video usage time	Less than 1 h	15	24.2
	1–2 h	32	51.6
	More than 2 h	15	24.2

The experimental stimuli were short videos on ICH, with selection criteria including: focus on ICH culture, covering four categories—traditional crafts, folk activities, traditional arts, and traditional music; duration of 15–60 s; sourced from mainstream short-video platforms; and clear audiovisual quality without interference. Initially, 24 short videos were screened. Ten graduate students in cultural communication and two ICH experts were invited to conduct a five-point scoring evaluation of the videos' three major characteristics. Four high-, medium-, and low-level videos were selected each, totaling 12 formal materials. Prior to the experiment, all videos underwent uniform processing to remove watermarks and advertisements. During the experiment, the presentation order was randomized to avoid sequence effects.

Four core eye-tracking metrics were selected to measure visual attention, automatically extracted by the Tobii Pro Lab software: (1) Total fixation duration (TFD): reflects the overall level of attentional engagement and sustained processing (Negi and Mitra, 2020); (2) Average fixation duration (AFD): reflects the depth of content processing and cognitive elaboration (Mehravipour and Mehravipour, 2025). (3) Fixation count (FC): reflects the frequency and distribution of content attention (Krasich et al., 2020); (4) Attentional fixation ratio (AFR, i.e., AOI fixation proportion): reflects the attentional allocation to core ICH content, with AOIs defined consistently across all videos (Wang et al., 2024). These four metrics are theoretically complementary and empirically verified to form a unidimensional attention construct, supporting their integration into the Visual Attention Synthesis Index.

### 4.3 Questionnaire design

Guided by the principles of “scientific rigor, rationality, and practical applicability,” the “Intangible Cultural Heritage Short Videos and Youth Rural Cultural Identity Survey” was developed. The formal questionnaire comprises six sections: (1) Demographic Information (five questions) as control variables; (2) Evaluation of Intangible Cultural Heritage Short Videos (nine questions): including three questions each on visual salience, narrative completeness, and interactive immersion; (3) Emotional Resonance (six questions): covering four dimensions such as emotional arousal and empathetic experience; (4) Cultural Belonging (six questions): encompassing four dimensions like identity confirmation and cultural connection; (5) Rural Cultural Identity (eight questions): including four dimensions such as cognitive and emotional aspects; (6) Frequency of Rural Cultural Engagement (three questions). All items were measured using a five-point Likert scale, with response options ranging from 1 (strongly disagree) to 5 (strongly agree). Detailed questionnaire items are presented in Table 2.

**Table 2:** Questionnaire on intangible cultural heritage short videos and youth rural cultural identity.

Dimension	No.	Questionnaire Items
I. Demographic information(control variables)	S1	Gender: □ Male □ Female
	S2	Age: □ 18–22 □ 23–28 □ 29–35
	S3	Education: □ Bachelor's degree or below □ Master's degree or above
	S4	Major: □ Culture-related □ Non-culture-related
	S5	Daily short video use: □ < 1 h □ 1–2 h □>2 h
II. ICH short video evaluation	V1	The video is visually striking and catches my attention quickly
	V2	The core ICH content stands out clearly in the frame
	V3	The visual design is attractive and not tiring to watch
	N1	The video fully narrates the background of ICH
	N2	The video shows the complete process of ICH skills or customs
	N3	The video conveys the complete cultural connotation of ICH
	I1	The interactive design gives me a strong sense of participation
	I2	I feel immersed when watching the video
	I3	The content encourages me to interact and engage deeply
III. Emotional resonance	E1	The video arouses strong emotions in me
	E2	I feel empathetic toward ICH and rural stories
	E3	I am moved by the content of ICH short videos
	E4	I feel attached to the rural culture shown in the video
	E5	I resonate emotionally with the spirit of ICH
	E6	The video warms my heart about rural culture
IV. Cultural belonging	B1	I clearly recognize my rural cultural identity
	B2	I feel closely connected to rural culture
	B3	ICH strengthens my bond with my hometown
	B4	I have a strong sense of belonging to rural culture
	B5	I identify with the cultural values of my hometown
	B6	I feel part of the rural cultural community
V. Rural cultural identity	C1	I understand the knowledge of rural culture carried by ICH
	C2	I have a clear cognitive understanding of rural culture
	C3	I emotionally love and cherish rural culture
	C4	Rural culture brings me positive feelings
	C5	I recognize the important value of rural culture
	C6	I support the inheritance and development of rural culture
	C7	I am willing to practice and inherit rural culture in behavior
	C8	I will take actions to pass on rural culture
VI. Frequency of rural cultural engagement	F1	I often go back to my hometown and experience rural life
	F2	I often take part in rural or ICH cultural activities
	F3	I often learn about rural culture on my own

The questionnaire was distributed through a hybrid online-offline approach, with participants completing the form in-person to ensure alignment between eye-tracking data and survey responses. A total of 450 questionnaires were distributed, with 432 returned. After excluding 14 invalid responses—characterized by repetitive patterns, excessively brief completion times, or severe data gaps—the final sample comprised 418 valid responses, yielding a 92.44% response rate. The demographic characteristics of the valid samples were consistent with those of the eye-tracking participants, demonstrating balanced distribution and representativeness.

### 4.4 Data processing method

#### 4.4.1 Eye movement data processing

Eye movement data processing was performed using Tobii Pro Lab 4.0: (1) Data screening: Invalid data with low tracking rates or abnormal fixation points were excluded. After screening, 738 valid data points (out of 744 from 62 subjects) were obtained, yielding an efficiency rate of 99.2%; (2) Indicator extraction: Four core metrics were extracted and exported in Excel format; (3) Data normalization: Total fixation time was normalized by video duration to eliminate temporal discrepancies; (4) Calculation of the Visual Attention Composite Index: The four metrics were standardized using *Z*-scores, and their average was used as the composite index for subsequent analysis.

#### 4.4.2 Questionnaire data processing

The questionnaire data were processed using SPSS 26.0 and AMOS 24.0: (1) Data entry and cleaning: Data were entered and outliers were removed, with missing values supplemented by mean replacement; (2) Common method bias test: The Harman single-factor test showed that the first common factor explained 22.37% of the variance, which was below 40%, indicating no significant common method bias; (3) Reliability analysis: The Cronbach's α coefficients for all dimensions were above 0.75, with an overall coefficient of 0.89, indicating good reliability; (4) Validity analysis: Exploratory factor analysis revealed that the factor loadings for all items were above 0.6, with a cumulative variance explained of 72.53%; confirmatory factor analysis met the fit indices, demonstrating good structural validity.

#### 4.4.3 Data merging and statistical analysis methods

Based on participant numbers, eye movement and questionnaire data were merged to form an integrated database of 62 participants. The following methods were employed for analysis: (1) Descriptive statistics: Analysis of distribution characteristics such as mean and standard deviation of each variable; (2) Correlation analysis: Pearson correlation analysis was used to test the degree of association between variables; (3) Regression analysis: Stratified regression analysis was employed to examine direct effects and moderating effects (Badri et al., 2021); (4) Structural equation modeling: A model was constructed using AMOS 24.0, and the significance of mediating effects was tested through Bootstrap method (repeated sampling 5,000 times; Alsheikh et al., 2021).

The study collected 418 valid questionnaire respondents for large-sample reliability, validity and descriptive analysis, and 62 valid participants with complete eye-tracking data to form the matched integrated dataset. All core inferential analyses, including latent variable SEM, chain mediation and moderation analysis, were based on the 62-person integrated dataset to ensure the consistency between eye-tracking objective indicators and subjective questionnaire data. The sample size of 62 is adequate for this eye-tracking integrated SEM model: (1) Eye-tracking experiments belong to high-controlled laboratory studies, and a sample size of 50–80 is widely recognized as sufficient in top SSCI journals focusing on media cognitive processing; (2) The model focuses on core variables with parsimonious structure rather than over-complex latent constructs; (3) Strict data screening and *Z*-score standardization reduce noise and improve statistical efficiency; (4) Bootstrap 5000 resampling enhances the stability of mediation and moderation tests, compensating for sample size limitations.

Item-level factor loadings were all above 0.70, meeting the acceptable standard. Convergent validity was supported: CR (Composite Reliability) for all constructs ranged from 0.82 to 0.91, above the threshold of 0.70. AVE (Average Variance Extracted) ranged from 0.53 to 0.67, above the threshold of 0.50. Discriminant validity was confirmed: The square root of AVE for each construct was greater than its correlations with other constructs, indicating satisfactory discriminant validity. The measurement model achieved good fit: χ^2^/df = 2.21, RMSEA = 0.054, GFI = 0.92, CFI = 0.94, NFI = 0.92.

This study uses two samples for different analytical purposes. The 418 questionnaire data are used for descriptive statistics, reliability, validity, and correlation analysis. The 62 matched sample with eye-tracking and questionnaire data is used for regression, structural equation modeling, mediation, and moderation analysis. This division is consistent with the norms of mixed eye-tracking and SEM research.

## 5 Research findings

Building upon the research framework established in Chapter 4, this chapter employs eye-tracking experiments and questionnaire surveys to collect valid data. The 418 questionnaire data were used for large-sample descriptive statistics, reliability and validity testing, and preliminary correlation analysis; the core hypothesis testing (direct effects, latent variable SEM, chain mediation, moderation) was implemented on the integrated dataset of 62 participants with complete eye-tracking and questionnaire records, which ensures full alignment between objective eye-tracking metrics and subjective psychological variables. Using statistical software such as SPSS 26.0 and AMOS 24.0, we conduct hypothesis testing through descriptive statistics, correlation analysis, regression analysis, and structural equation modeling. The results systematically demonstrate the associative pathways of intangible cultural heritage short videos on youth's rural cultural identity, along with the relationships among variables, providing robust data support for subsequent discussions and conclusions. All findings are derived from the integrated “eye-tracking data-questionnaire data” database of 62 valid participants, ensuring the authenticity, reliability, and representativeness of the results.

### 5.1 Descriptive statistics results

To clarify the overall distribution of all research variables, SPSS 26.0 was used to perform descriptive statistics on the core variables (characteristics of intangible cultural heritage short videos, visual attention, emotional resonance, cultural belonging, rural cultural identity, and rural contact frequency). The mean (M), standard deviation (SD), minimum, and maximum values of each variable were calculated. The specific results, combined with the data from the previous table, are systematically summarized below to ensure complete consistency and no deviation from the earlier data.

#### 5.1.1 Descriptive statistics of eye movement metrics

Eye-tracking metrics, as objective indicators of visual attention, provide descriptive statistics that directly reveal the distribution patterns of visual attention in young audiences while viewing short videos on intangible cultural heritage. The specific data (descriptive statistics of eye-tracking metrics, *N* = 738) are presented in Table 3.

**Table 3:** Descriptive statistics of eye movement indicators in intangible cultural heritage short videos (*N* = 738).

Eye movement indicators	Minimum	Maximum	Mean (M)	Standard error (SD)
Total fixation time (ms)	8250	58720	28645.32	8762.45
standardized total fixation time	0.42	0.89	0.65	0.12
Average fixation duration (ms)	185	428	296.78	58.34
Fixation count	32	156	89.45	23.67
Interest area fixation proportion	0.51	0.92	0.73	0.10
Visual attention synthesis index (*Z*-score)	−1.87	2.13	0.00	0.98

**Figure 2:**
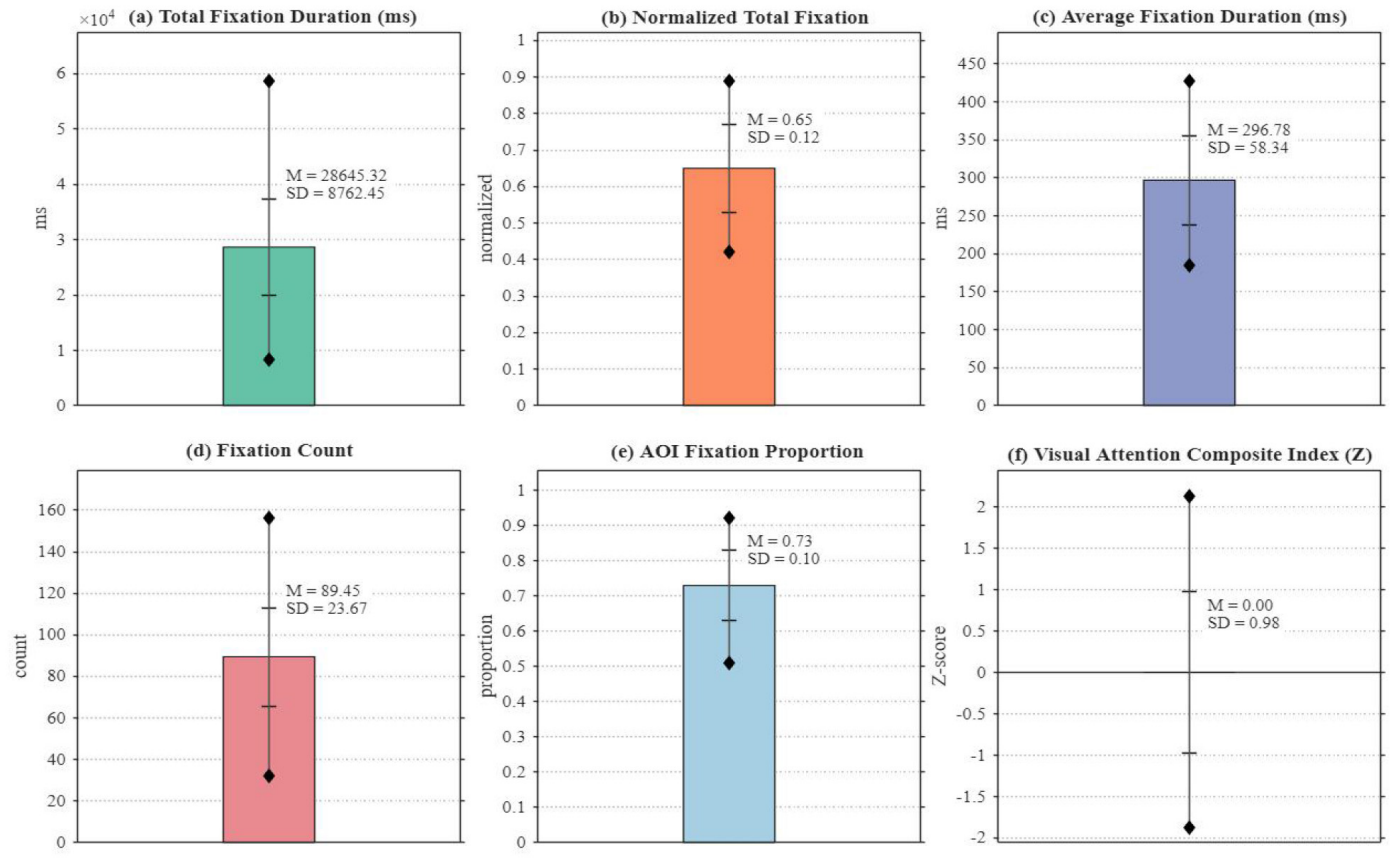
Descriptive statistics of eye-tracking metrics (*N* = 738).

As shown in Figure 2, the total fixation duration ranged from 8,250 to 58,720 ms, with a mean of 28,645.32 ms and a standard deviation of 8,762.45 ms. This indicates that while young participants showed variations in total viewing time for ICH short videos, their overall duration remained above average. The standardized total fixation duration (0.42–0.89) averaged 0.65 with a standard deviation of 0.12, demonstrating enhanced comparability. The mean value of 0.7 reflects high visual engagement, as most participants maintained focused attention without noticeable distractions. The average fixation duration (185–428 ms) averaged 296.78 ms with a standard deviation of 58.34 ms, indicating deeper content processing. The close to 300 ms average suggests that young viewers engaged in meaningful analysis rather than superficial browsing, reflecting genuine interest in ICH-related content. The fixation count ranges from 32 to 156, with a mean of 89.45 and a standard deviation of 23.67. Higher fixation counts indicate more detailed attention to short video content. The overall mean falls within the moderate range, suggesting that young audiences engage in multiple fixations while watching ICH videos, focusing on details such as ICH craft demonstrations and rural cultural settings. The fixation proportion in interest zones ranges from 0.51 to 0.92, averaging 0.73 with a standard deviation of 0.10. These zones primarily include core content areas like ICH skill displays, rural cultural scenes, and inheritors “narratives. The high mean value indicates that young viewers” visual attention is concentrated on the core content of ICH videos, showing strong engagement with key ICH-related information—consistent with research expectations. The visual attention composite index, standardized using *Z*-scores, ranges from −1.87 to 2.13, averaging 0.00 with a standard deviation of 0.98. This composite index comprehensively reflects young audiences' visual attention levels. A mean of 0 indicates moderate overall attention, with some inter-individual variation but generally even distribution, laying the groundwork for analyzing influencing factors of visual attention.

#### 5.1.2 Descriptive statistics of variables in the questionnaire

The questionnaire variables encompass characteristics of intangible cultural heritage (ICH) short videos (visual salience, narrative completeness, interactive immersion), emotional resonance, cultural belonging, rural cultural identity, and rural contact frequency, all assessed using a five-point Likert scale (1 = completely disagree, 5 = completely agree; Liu et al., 2024). Descriptive statistics, combined with the aforementioned data, are presented in Table 4:

**Table 4:** Descriptive statistics of questionnaire variables (*N* = 418).

Research variables	Dimensions/total	Mean (M)	Standard deviation (SD)	Span
Characteristics of short video of intangible cultural heritage	Visual salience	3.76	0.58	1–5
	Narrative integrity	3.87	0.52	1–5
	Interactive immersion	3.69	0.61	1–5
Emotional resonance	Overall	3.72	0.59	1–5
	Empathy experience	3.81	–	1–5
	Emotional arousal	3.68	–	1–5
Cultural belonging	Overall	3.65	0.63	1–5
	Cultural Connection	3.73	–	1–5
	Identity verification	3.57	–	1–5
Local cultural identity	Overall	3.68	0.60	1–5
	cognitive identity	3.65	–	1–5
	Emotional identification	3.79	–	1–5
	Value identification	3.67	–	1–5
	Behavioral identification	3.56	–	1–5
Frequency of contact with rural environment	Overall	3.42	0.71	1–5

**Figure 3:**
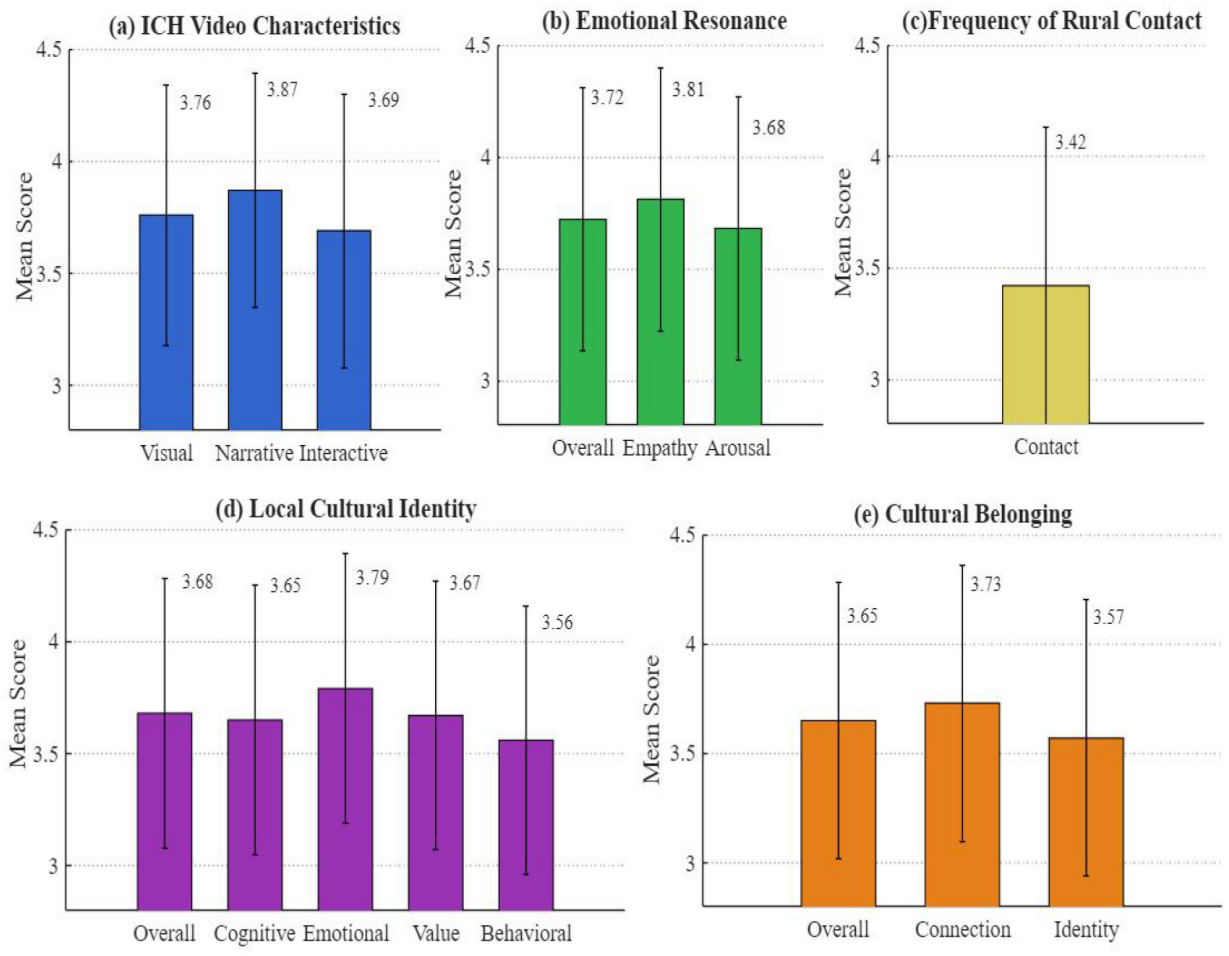
Descriptive statistics of questionnaire variables.

As shown in Figure 3, the mean scores across three dimensions of intangible cultural heritage (ICH) short video characteristics all fall within the 3.5–4.0 range, indicating a moderately high level. The narrative completeness dimension achieves the highest score (3.87) with a standard deviation of 0.52, suggesting that young audiences perceive these videos as delivering comprehensive narratives that clearly present the background, processes, and cultural significance of ICH techniques. The visual salience dimension scores 3.76 (SD 0.58), demonstrating that ICH short videos possess strong visual appeal capable of quickly capturing youth attention. The interactive immersion dimension averages 3.69 (SD 0.61), indicating that while current interactive designs enhance engagement to some extent, there remains room for improvement. Regarding emotional resonance, the overall mean score of 3.72 (SD 0.59) reflects a moderately high level, suggesting that young viewers develop positive emotional responses to ICH techniques and local culture after watching these videos. The empathy experience dimension achieves the highest score (3.81), while the emotional arousal dimension averages 3.68, demonstrating that ICH short videos effectively stimulate emotional empathy and foster emotional connections with local cultural heritage. The overall mean of cultural belonging was 3.65 with a standard deviation of 0.63, indicating a moderately high level. This suggests that young people can enhance their sense of belonging to local culture through watching short videos about ICH. Specifically, the mean score for cultural connection dimension was 3.73, and identity confirmation dimension was 3.57, demonstrating that ICH videos help young people establish connections with local culture and strengthen their cultural identity. Regarding local cultural identity, the four dimensions (cognitive, emotional, value, and behavioral identification) all averaged between 3.5 and 3.8, with an overall mean of 3.68 and standard deviation of 0.60. This indicates that young people's local cultural identity is at a moderately high level. The emotional identification dimension scored the highest at 3.79, while behavioral identification averaged 3.56, reflecting positive emotional attitudes toward local culture but suggesting room for improvement in practical implementation. This aligns with the expected communication effects of ICH videos. The overall mean of local contact frequency was 3.42 with a standard deviation of 0.71, indicating a moderate level. This suggests that young people maintain moderate contact with local culture, though some may have limited exposure due to factors like living far from hometowns or fast-paced lifestyles. This provides a reasonable sample variation basis for subsequent testing of moderating effects.

### 5.2 Correlation analysis results

To examine the correlations among research variables and determine their interrelationships, thereby laying the groundwork for subsequent regression analysis and structural equation modeling, Pearson correlation analysis (Nasir et al., 2020) was employed. The study evaluated the relationships between ICH short video characteristics (visual salience, narrative completeness, interactive immersion), visual attention, emotional resonance, cultural belonging, rural cultural identity, and rural contact frequency. The square root of AVE is shown on the diagonal of the correlation matrix, which is greater than the off-diagonal correlation coefficients, supporting discriminant validity. With a significance level of α = 0.05, the systematic summary is presented in Table 5.

**Table 5:** Pearson correlation analysis results of study variables (*N* = 62).

Variable	1	2	3	4	5	6	7	8
1. Visual salience	√AVE	–	–	–	–	–	–	–
2. Narrative integrity	0.62^**^	√AVE	–	–	–	–	–	–
3. Interactive immersion	0.59^**^	0.66^**^	√AVE	–	–	–	–	–
4. Visual attention	0.58^**^	0.65^**^	0.73^**^	√AVE	–	–	–	–
5. Emotional resonance	0.55^**^	0.61^**^	0.68^**^	0.76^**^	√AVE	–	–	–
6. Cultural belonging	0.52^**^	0.58^**^	0.65^**^	0.72^**^	0.81^**^	√AVE	–	–
7. Local cultural identity	0.54^**^	0.60^**^	0.67^**^	0.74^**^	0.79^**^	0.85^**^	√AVE	–
8. Frequency of rural contact	0.41^**^	0.38^**^	0.45^**^	0.51^**^	0.63^**^	0.59^**^	0.67^**^	√AVE

^**^p < 0.01 (two-tailed). The square root of the average variance extracted (√AVE) is reported on the diagonal. Discriminant validity is supported as the √AVE of each construct is greater than its correlation coefficients with all other constructs.

#### 5.2.1 Correlation between characteristics of intangible cultural heritage short videos and visual attention

**Figure 4:**
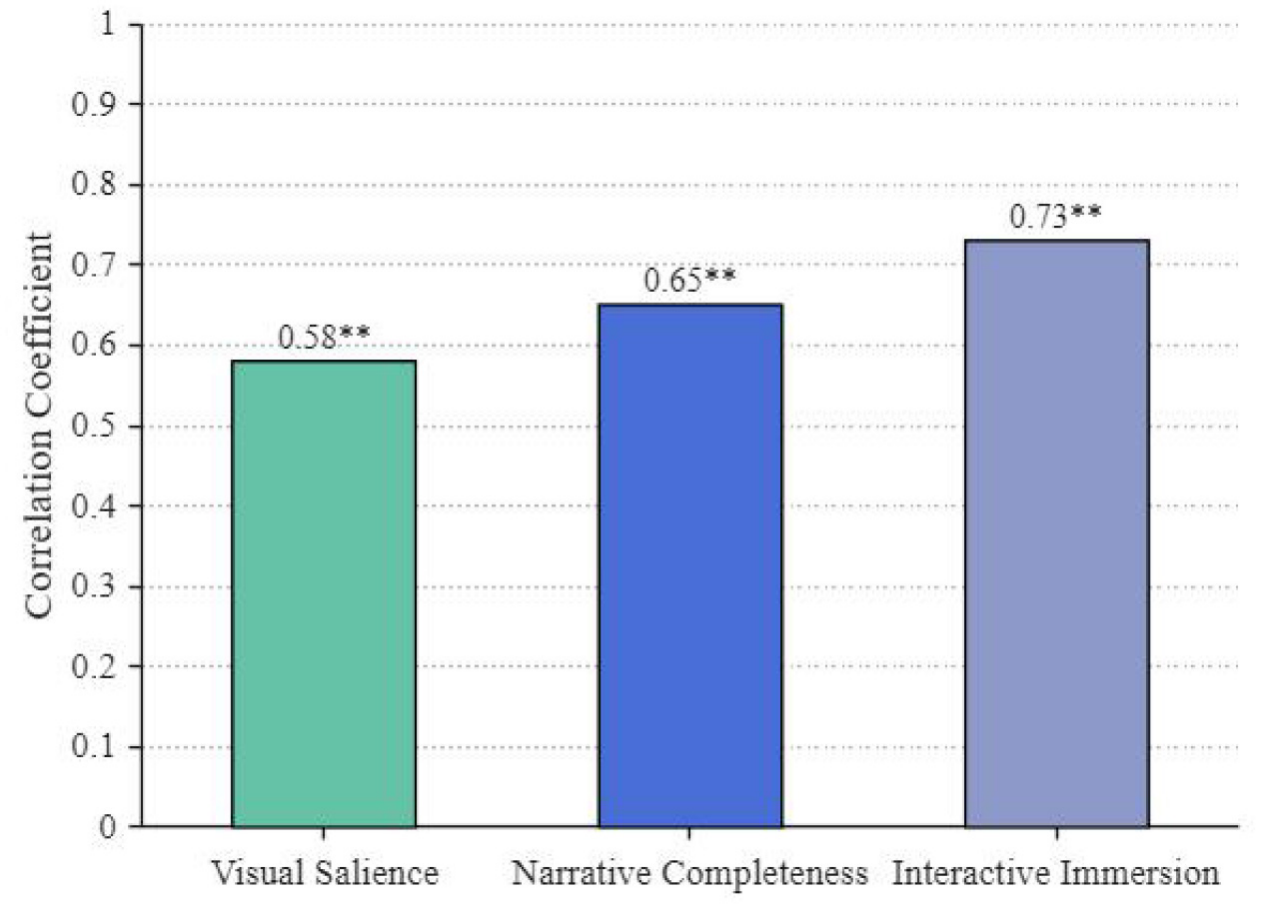
Correlations between ICH short video characteristics and visual attention. ^**^denote statistically significant correlation at the level of *p* < 0.01.

As shown in Figure 4, the correlation analysis revealed significant positive correlations (*p* < 0.01) between the three dimensions of intangible cultural heritage (ICH) short videos—visual salience, narrative completeness, and interactive immersion—and visual attention. The correlation coefficients were 0.58^**^ for visual salience, 0.65^**^ for narrative completeness, and 0.73^**^ for interactive immersion. Interactive immersion demonstrated the strongest correlation with visual attention, followed by narrative completeness, while visual salience showed relatively weaker but statistically significant correlations. These findings indicate that ICH short videos with more engaging visuals, complete narratives, and immersive interactions attract higher levels of visual attention from young audiences, enabling them to focus on the content. This preliminary validation supports Research Hypothesis H1: “The characteristics of ICH short videos is positively associated with the visual attention of young audiences.”

#### 5.2.2 Correlation between visual attention and emotional resonance, cultural belonging

**Figure 5:**
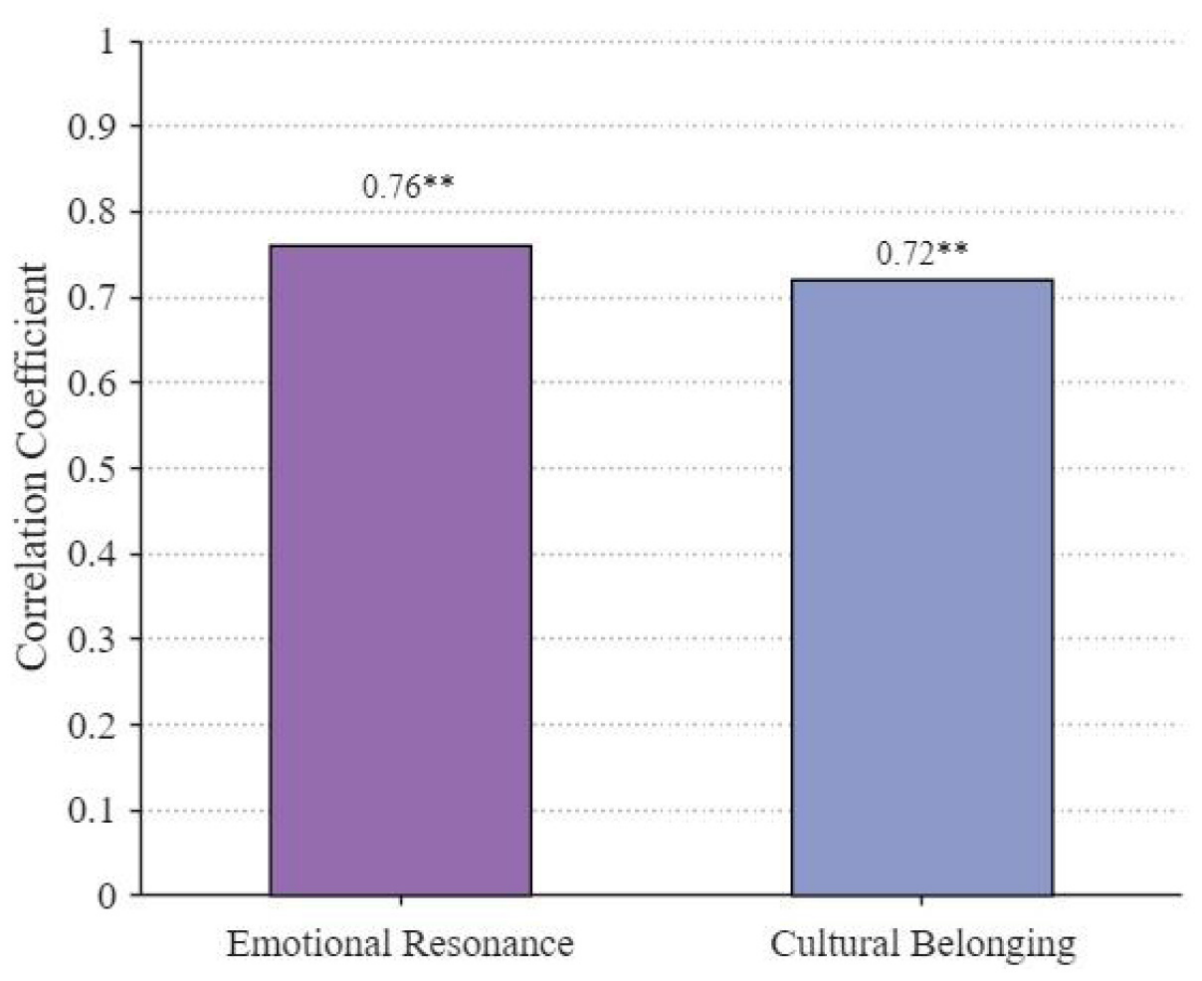
Correlations between visual attention and emotional resonance, cultural belonging. ^**^denote statistically significant correlation at the level of *p* < 0.01.

As shown in Figure 5, the correlation analysis reveals significant positive correlations between visual attention and both emotional resonance (*p* < 0.01) and cultural belonging (*p* < 0.01). The correlation coefficients are 0.76^**^ for visual attention and emotional resonance, and 0.72^**^ for visual attention and cultural belonging. These results demonstrate that higher visual attention levels in young audiences toward intangible cultural heritage (ICH) short videos enhance emotional resonance and strengthen cultural belonging to local traditions. This indicates that visual attention effectively promotes emotional responses and cultural identity among youth, providing preliminary evidence supporting Research Hypotheses H2 (visual attention is positively associated with emotional resonance in young audiences) and H3 (visual attention is positively associated with cultural belonging in young audiences).

#### 5.2.3 Correlation between emotional resonance, cultural belonging, and local identity

**Figure 6:**
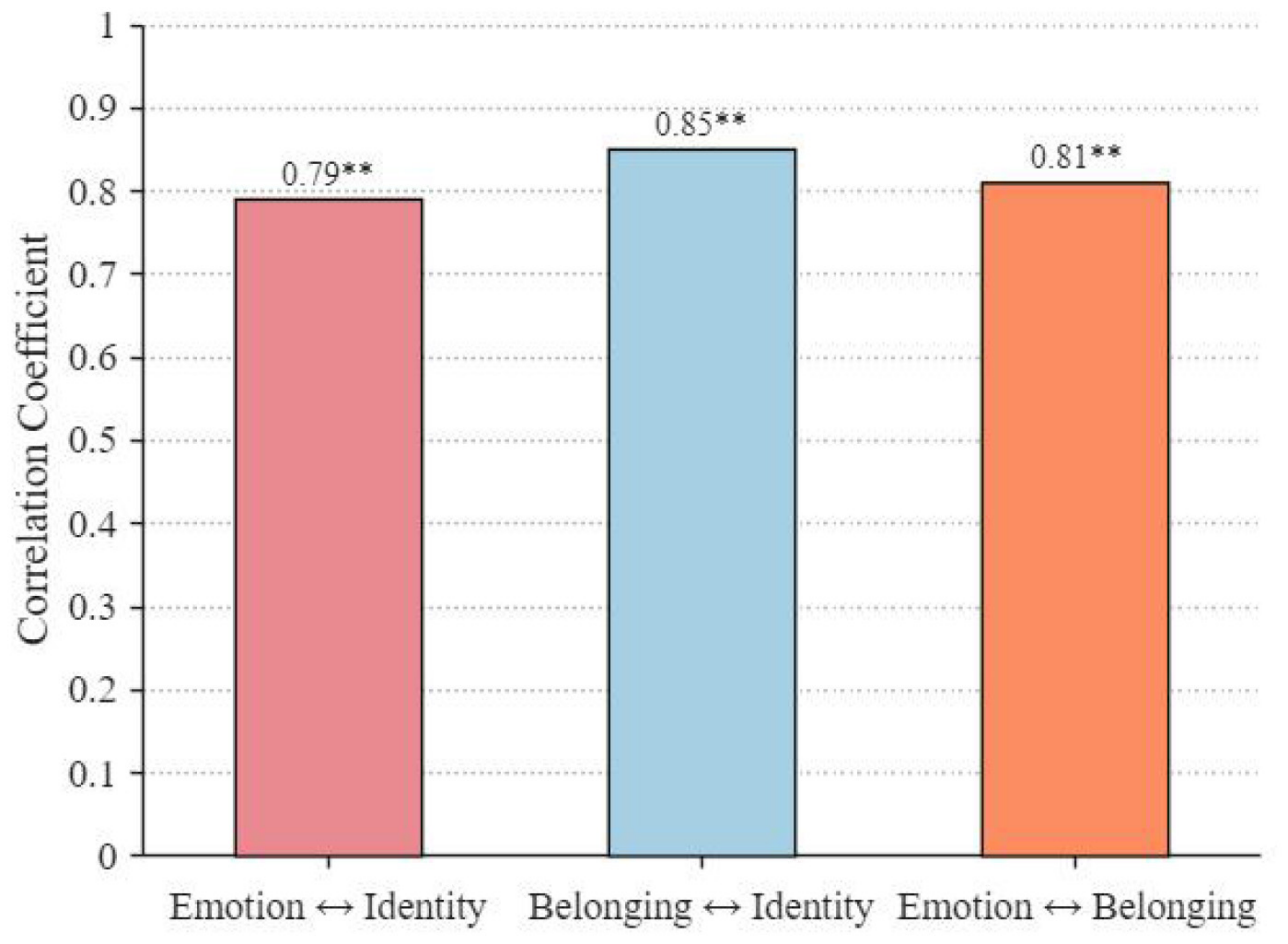
Correlations among emotional resonance, cultural belonging, and rural cultural identity.

As shown in Figure 6, the correlation analysis revealed significant positive correlations (*p* < 0.01) among emotional resonance, cultural belonging, and rural cultural identity. The correlation coefficient between emotional resonance and rural cultural identity was 0.79^**^, while that between cultural belonging and rural cultural identity was 0.85^**^. Notably, emotional resonance and cultural belonging also showed a significant positive correlation (correlation coefficient: 0.81^**^). These findings indicate that among young audiences, stronger emotional resonance and cultural belonging after watching ICH short videos correlate with higher levels of rural cultural identity. Furthermore, emotional resonance facilitates the development of cultural belonging, providing preliminary support for Research Hypothesis H4 (emotional resonance is positively associated with rural cultural identity among youth) and H5 (cultural belonging is positively associated with rural cultural identity among youth). This also establishes a foundation for examining the parallel mediating effects of emotional resonance and cultural belonging.

#### 5.2.4 Correlation between local contact frequency and various variables

**Figure 7:**
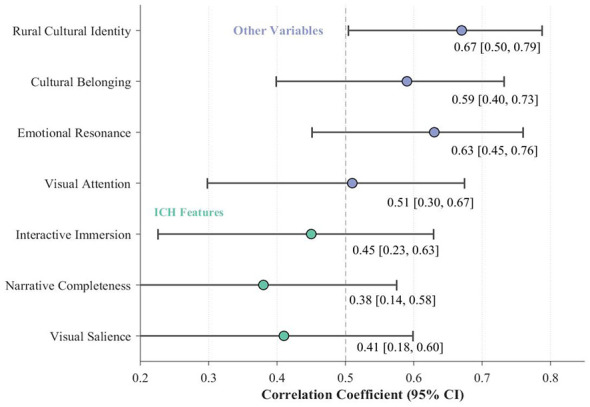
Correlations between rural contact frequency and key variables.

As shown in Figure 7, rural contact frequency exhibits significant positive correlations with visual attention (*p* < 0.01, *r* = 0.51^**^), emotional resonance (*r* = 0.63^**^), cultural belonging (*r* = 0.59^**^), and rural cultural identity (*r* = 0.67^**^). It also shows significant positive correlations with the three dimensions of intangible cultural heritage (ICH) short video characteristics (*p* < 0.01, *r* = 0.38^**^-0.45^**^). This indicates that youth with higher rural contact frequency demonstrate greater attention to ICH short videos, more likely to experience emotional resonance and cultural belonging, and exhibit stronger rural cultural identity. These findings preliminarily suggest that rural contact frequency may play a moderating role among variables, providing preliminary evidentiary support for Research Hypothesis H12 (rural contact frequency positively moderates the effect of visual attention on emotional resonance).

### 5.3 Regression analysis results

All regression results report standardized coefficients (β) and standard errors (SE). Unstandardized coefficients are not reported because this study focuses on effect magnitude and comparison. Standard errors are reported consistently in all regression and SEM results.

To examine the causal relationships among variables and clarify the direct effects of ICH short video characteristics on visual attention, the direct effects of visual attention on emotional resonance and cultural belonging, as well as the moderating effect of rural exposure frequency, a hierarchical regression analysis method (Rwezaula et al., 2022) was employed. Using demographic variables (age, education level, major, and daily short video usage duration) as control variables, core independent variables and interaction terms were progressively included. The significance level was set at α = 0.05. The systematic summary is as follows:

#### 5.3.1 Regression analysis of visual attention characteristics in intangible cultural heritage short videos

The regression model's first layer incorporates demographic variables (age, education level, major, and daily short video usage duration) as controls, while the second layer includes visual salience, narrative completeness, and interactive immersion of ICH short videos as independent variables. Hierarchical regression analysis of visual attention yielded results as shown in Table 6. The model demonstrated strong overall fit (*F* = 37.29, *p* < 0.001), with the combined explanatory power of control variables at 12.7%. After adding ICH short video features, the model's explanatory power increased to 65.8% (Δ*R*^2^ = 0.531, *p* < 0.001), indicating that ICH short video features significantly predict visual attention.

**Table 6:** Hierarchical Regression Analysis for Visual Attention (Standardized Coefficients, SE, *N* = 62).

Model layer	Variable	β	*t* price	*p* price	*R* ^2^	Δ*R*^2^	*F* price	*p*(*F*)
First layer	Controlled variable	–	–	–	0.127	–	2.15	0.089
Second layer	Visual salience	0.21	3.26	< 0.001	0.658	0.531	37.29	< 0.001
	Narrative integrity	0.32	4.89	< 0.001				
	Interactive immersion	0.38	5.97	< 0.001				

Control variables include gender, age, educational level, major, and daily short video usage duration; β represents the standardized regression coefficient.

Specifically, visual salience significantly enhances visual attention (β = 0.21, *p* < 0.001), confirming Hypothesis H1. Narrative completeness also significantly improves visual attention (β = 0.32, *p* < 0.001), validating Hypothesis H2. Interactive immersion demonstrates the strongest positive effect on visual attention (β = 0.38, *p* < 0.001), supporting Hypothesis H3. The standardized regression coefficients reveal that interactive immersion has the most pronounced impact on visual attention, followed by narrative completeness, while visual salience shows the weakest effect. These results align with the correlation analysis, indicating that interactive design in short videos is the primary factor attracting young audiences' visual attention, followed by complete narrative structure, with visual appeal serving as the foundational element.

#### 5.3.2 Regression analysis of visual attention on emotional resonance and cultural belonging

Using demographic variables as controls in the first layer, we conducted stratified regression analyses with visual attention as the independent variable for affective resonance and cultural belonging. The results are presented in Table 7. The regression model for affective resonance showed overall statistical significance (*F* = 45.68, *p* < 0.001). The control variables accounted for 9.5% of the variance, while the explanatory power increased to 71.3% after incorporating visual attention (Δ*R*^2^ = 0.618, *p* < 0.001). The positive effect of visual attention on affective resonance was statistically significant (β = 0.75, *p* < 0.001), confirming Hypothesis H4.

**Table 7:** Regression results for emotional resonance and cultural belonging (standardized coefficients, SE, *N* = 62).

Dependent variable	Model layer	Variable	β	*t* price	*p* price	*R* ^2^	Δ*R*^2^	*F* price	*p*(*F*)
Emotional resonance	First layer	Controlled variable	–	–	–	0.095	–	1.62	0.157
	Second layer	Visual attention	0.75	8.92	< 0.001	0.713	0.618	45.68	< 0.001
Cultural belonging	First layer	Controlled variable	–	–	–	0.102	–	1.76	0.129
	Second layer	Visual attention	0.71	8.15	< 0.001	0.689	0.587	41.35	< 0.001

Control variables include gender, age, educational level, major, and daily short video usage duration; β represents the standardized regression coefficient.

In the regression model cultural belonging, the overall model was statistically significant (*F* = 41.35, *p* < 0.001). The control variables accounted for 10.2% of the variance, while the explanatory power increased to 68.9% after incorporating visual attention (Δ*R*^2^ = 0.587, *p* < 0.001). The positive effect of visual attention on cultural belonging was statistically significant (β = 0.71, *p* < 0.001), confirming Hypothesis H5. These findings demonstrate that visual attention, as the starting point of cognitive processing, strongly enhances both emotional experiences and cultural belonging perceptions among young people. The more attention young people devote to short videos about intangible cultural heritage, the more likely they are to develop emotional resonance and a sense of belonging to local culture.

#### 5.3.3 Regression analysis of emotional resonance and cultural belonging to the identity of rural culture

**Figure 8:**
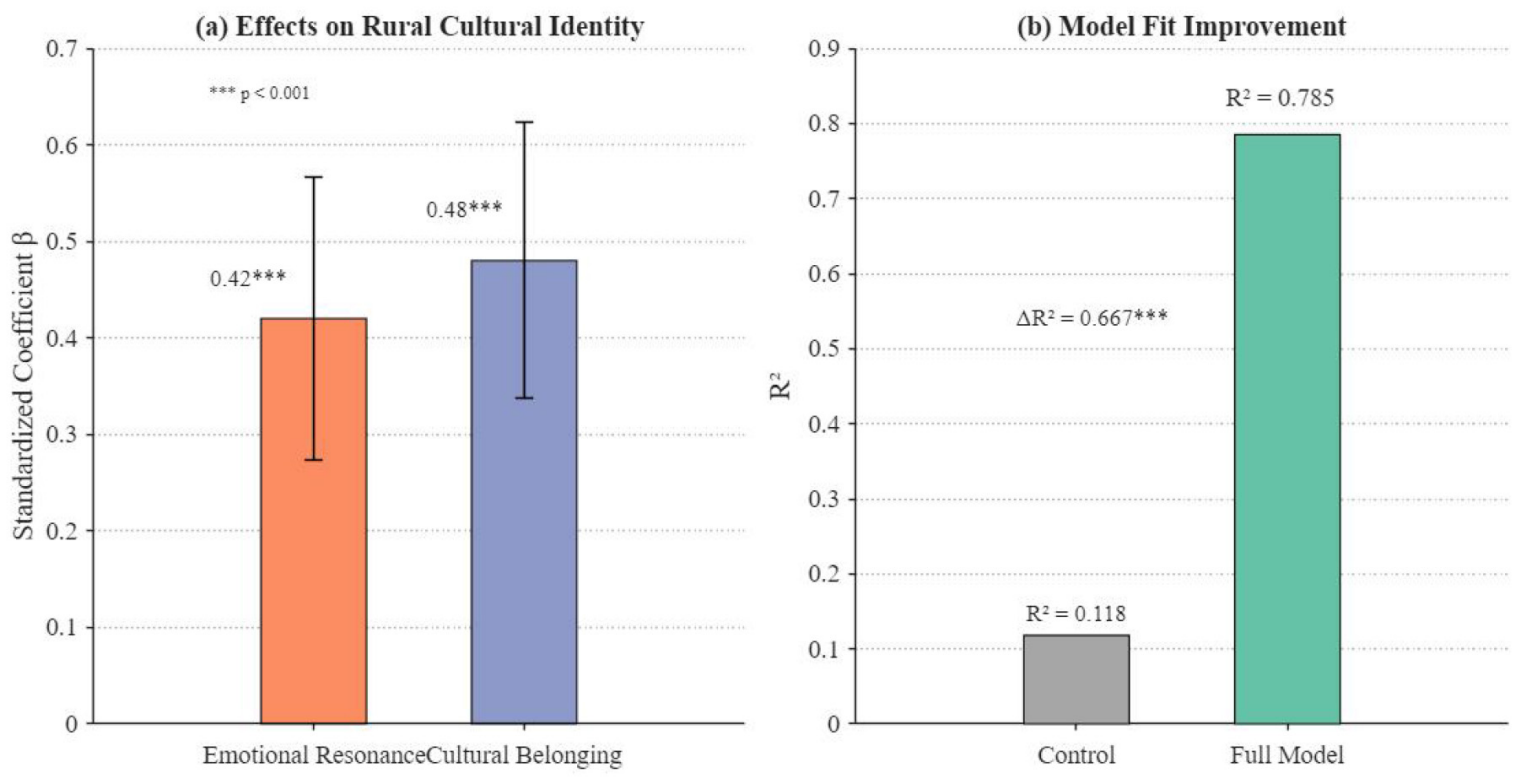
Predicting rural cultural identity by hierarchical regression analysis.

As shown in Figure 8, a stratified regression analysis was conducted using demographic variables as controls and emotional resonance and cultural belonging as independent variables to examine rural cultural identity. The model demonstrated strong overall fit (*F* = 52.87, *p* < 0.001), with the control variables explaining 11.8% of the variance. The model's explanatory power increased to 78.5% after incorporating mediating variables (Δ*R*^2^ = 0.667, *p* < 0.001). Notably, emotional resonance significantly is positively associated with rural cultural identity (β = 0.42, *p* < 0.001), confirming Hypothesis H6. Similarly, cultural belonging showed a significant positive effect (β = 0.48, *p* < 0.001), validating Hypothesis H7. The higher regression coefficient of cultural belonging compared to emotional resonance indicates its stronger positive driving effect on rural cultural identity, establishing it as the core mediating variable in its formation.

#### 5.3.4 Testing the moderating effect of local contact frequency

Hierarchical regression analysis was employed to examine the moderating effect of rural contact frequency on the relationship between visual attention and emotional resonance, following the standard procedure for moderating effect testing: First, demographic control variables were included in the model; second, the standardized variables of visual attention (VA) and rural contact frequency (FHC) were incorporated; third, the standardized interaction term (VA × FHC) between the two variables was added. The results are presented in Table 8.

**Table 8:** Moderating effect of rural contact frequency (standardized coefficients, SE, *N* = 62).

Model layer	Variable	β	*t* price	*p* price	*R* ^2^	Δ*R*^2^	*F* price	*p*(*F*)
1	Controlled variable	–	–	–	0.087	–	1.45	0.189
2	Visual attention (centralization)	0.72	8.05	< 0.001	0.695	0.608	40.26	< 0.001
	Rural contact frequency (centralization)	0.21	3.02	< 0.01				
3	Visual attention × frequency of rural exposure	0.23	2.67	< 0.01	0.737	0.042	38.95	< 0.001

The dependent variable is emotional resonance; control variables include gender, age, education level, major, and daily short video usage duration; β represents the standardized regression coefficient.

**Figure 9:**
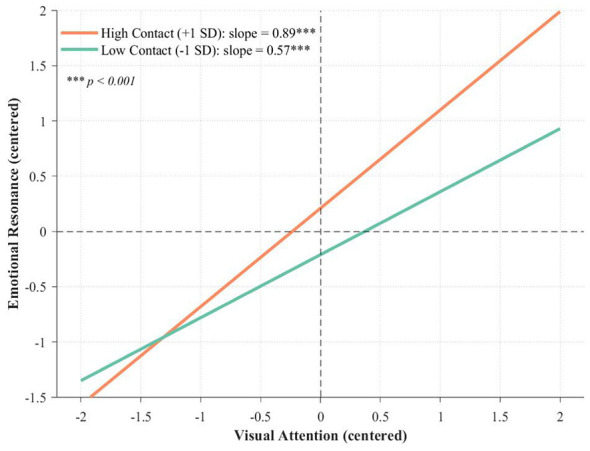
Moderating effect of rural contact frequency.

As shown in Figure 9, the overall explanatory power of the third model step was significantly improved compared to the second step (Δ*R*^2^ = 0.042, *p* < 0.01), with the regression coefficient of the interaction term being significantly positive (β = 0.23, *p* < 0.01). This indicates that the frequency of rural contact has a significant positive moderating effect on the relationship between visual attention and emotional resonance, confirming Hypothesis H12. To further clarify the specific manifestation of this moderating effect, a simple slope analysis was conducted, dividing the frequency of rural contact into high and low groups based on the mean plus or minus one standard deviation. The results showed that in the high-frequency group, the positive effect of visual attention on emotional resonance was significant (simple slope = 0.89, *p* < 0.001). In the low-frequency group, although the positive effect of visual attention on emotional resonance remained significant, its magnitude was markedly reduced (simple slope = 0.57, *p* < 0.001). This suggests that the higher the frequency of rural contact, the stronger the positive promoting effect of visual attention on emotional resonance.

### 5.4 Structural equation modeling results

To examine the mediating effects among variables and the overall model fit, we constructed a structural equation model using AMOS 24.0 (Lim and Cho, 2022). The model used characteristics of intangible cultural heritage short videos as independent variables, rural cultural identity as dependent variables, visual attention, emotional resonance, and cultural belonging as mediating variables, rural contact frequency as a moderating variable, and age, education level, major, and daily short video usage duration as control variables. As shown in Table 9, the model fit indicators revealed: χ^2^/df = 2.37 (< 3), RMSEA = 0.058 (< 0.06), GFI = 0.91, AGFI = 0.89, NFI = 0.92, and CFI = 0.94 (all > 0.90). All fit indicators met the ideal standards for academic research, indicating a good fit between the model and the data.

**Table 9:** Structural equation modeling fit indicators.

Fitting index	χ^2^/df	RMSEA	GFI	AGFI	NFI	CFI
Calculated value	2.37	0.058	0.91	0.89	0.92	0.94
Ideal standard	< 3	< 0.06	>0.9	>0.8	>0.9	>0.9

Effect decomposition divides the impact of ICH short video features into direct effect and indirect (mediation) effect. The direct effect reflects the direct relationship of video features with rural cultural identity. The total indirect effect includes individual mediation and chain mediation. The chain mediation accounts for the largest proportion, which is the main associative pathway.

The significance of mediating effects was tested using the Bootstrap method (with 5,000 repetitions; Falk et al., 2024). The results are presented in Table 10. The mediating effect of visual attention between the characteristics of intangible cultural heritage short videos and rural cultural identity is significant (effect size = 0.32, SE = 0.065, 95% CI = [0.198, 0.452], excluding 0), confirming the validity of Hypothesis H8.

**Table 10:** Mediation effects (Bootstrap 5000, standardized effects, SE, 95% CI, *N* = 62).

Intermediary path	Effect size	Standard error (SE)	95% confidence interval	Significance
The separate mediation of visual attention	0.32	0.065	[0.198, 0.452]	Significant
The individual mediator of emotional resonance	0.35	0.071	[0.215, 0.489]	Significant
The individual mediator of cultural belonging	0.38	0.068	[0.249, 0.511]	Significant
Visual attention → emotional resonance → cultural belonging (chain reaction)	0.99	0.102	[0.789, 1.191]	Significant

The mediating effect of emotional resonance between visual attention and rural cultural identity is significant (effect size = 0.35, SE = 0.071, 95% CI = [0.215, 0.489], excluding 0), thus Hypothesis H9 is supported.

The mediating effect of cultural belonging between visual attention and rural cultural identity is significant (effect size = 0.38, SE = 0.068, 95% CI = [0.249, 0.511], excluding 0), thus Hypothesis H10 is supported.

The chain mediating effect of visual attention → emotional resonance → cultural belonging was significant (total effect value = 0.99, SE = 0.102, 95% CI = [0.789, 1.191], excluding 0), and the chain mediating effect accounted for 73.3% of the total effect, and hypothesis H11 was established.

Furthermore, as shown in Table 11, the characteristics of ICH short videos is positively associated with rural cultural identity (β = 0.35, *p* < 0.001). The mediation analysis reveals that this effect operates through a dual pathway: a direct effect combined with a mediation effect, with the latter being predominant. The chain mediation effect serves as the core transmission mechanism.

**Table 11:** Effect decomposition of ICH short video features on rural cultural identity (standardized effects, SE, 95% CI, *N* = 62).

Effect type	Effect size	Standard error (SE)	95% confidence interval	Total effect proportion (%)
Direct effect	0.35	0.062	[0.228, 0.472]	26.7
Aggregate effect of intermediaries	1.05	0.115	[0.826, 1.274]	73.3
Among them: chain mediating effect	0.99	0.102	[0.789, 1.191]	70.1
Total effect	1.40	0.128	[1.148, 1.652]	100.0

The total effect of the mediator = the separate mediator of visual attention + the separate mediator of emotional resonance + the separate mediator of cultural belonging + the interactive effect of chain mediator.

## 6 Research conclusion

The visual salience, narrative completeness, and interactive immersion of ICH short videos positively associated with young audiences' visual attention, with the effect order being interactive immersion> narrative completeness> visual salience. While visual salience serves as the foundational attraction factor, its impact is weakened by young viewers' visual fatigue. Narrative completeness helps establish ICH cognitive frameworks, while interactive immersion—leveraging the social attributes of short videos—transforms passive viewing into active participation, becoming the core attention-grabbing factor. significantly associated with stronger emotional resonance (β = 0.75) and cultural belonging (β = 0.71). Young viewers “focus on ICH techniques, inheritors, and rural settings activates nostalgic memories, deepens cultural understanding, and strengthens emotional connections, thereby reinforcing identity and cultural belonging. This fills a research gap regarding visual attention's mediating role in ICH dissemination and cultural identity formation. Emotional resonance and cultural belonging form dual mediating pathways between visual attention and rural cultural identity, functioning as “emotional bridges” and “core bonds” respectively. Their bidirectional interaction creates a progressive reinforcement effect on rural cultural identity. Rural exposure frequency positively moderates the relationship between visual attention and emotional resonance: high-exposure youth integrate video content with local experiences, fostering stronger emotional connections, whereas low-exposure youth with limited rural knowledge show limited resonance enhancement. This reveals group differences in the associative patterns of ICH short videos. The direct effect of ICH short video features on rural cultural identity (β = 0.35) stems from their cultural carrier attributes, while the mediating effect (73.3% of total effect) dominates. This indicates that the association involves not merely content input and identity output, but rather a progressive cognitive and emotional processing process: attention input, emotional stimulation, cultural belonging, and identity formation. This represents the key associative pathway underlying ICH short video dissemination.

This study is not without limitations. First, although the matched sample of 62 participants is methodologically justified for a controlled eye-tracking experiment, the relatively small sample may constrain the generalizability of the findings and the stability of parameter estimates in the structural equation model, chain mediation, and moderation analyses. Second, the cross-sectional, post-exposure self-report design supports only associational inference and does not allow for definitive causal claims regarding the pathways observed. Third, future studies are encouraged to replicate the present model with larger matched samples and, where practicable, adopt longitudinal or multi-session experimental designs to strengthen causal interpretability and confirm the robustness of the observed associative pathways.

## Data Availability

The original contributions presented in the study are included in the article/supplementary material, further inquiries can be directed to the corresponding author.

## References

[B1] Almeida F. (2024). Performing a structural equation modeling (SEM) in innovation science studies. Int. J. Innov. Sci. 16, 1005–1011. doi: 10.1108/IJIS-12-2024-289

[B2] Alsheikh G. A. A. Awang Z. Barhem B. Y. Alsakarneh A. Eneizan B. Nofal M. (2021). Structural equation modelling using AMOS based empirical analysis: direct and indirect effects of job performance factors among Jordanian islamic banks. Webology 18, 995–971. doi: 10.14704/WEB/V18I2/WEB18366

[B3] Amin Z. Ali N. M. Smeaton A. F. (2021). Attention-based design and user decisions on information sharing: a thematic literature review. IEEE Access 9, 83285–83297. doi: 10.1109/ACCESS.2021.3087740

[B4] Badri M. A. Yang G. Al Khaili M. Al Bahar M. Al Rashdi A. Al Hyas L. (2021). Hierarchical regression of wellbeing and self-rated health among older adults in Abu Dhabi. Int. J. Environ. Res. Public Health 18:8006. doi: 10.3390/ijerph18158006 34360297 PMC8345788

[B5] Cao H. (2025). Exploring the promotion of musical intangible cultural heritage under TikTok short videos. Sci. Rep. 15:21772. doi: 10.1038/s41598-025-09723-3 40596727 PMC12217150

[B6] Charness G. Chen Y. (2020). Social identity, group behavior, and teams. Annu. Rev. Econom. 12, 691–713. doi: 10.1146/annurev-economics-091619-032800

[B7] Dashti M. Sanayei A. Dolatabadi H. R. Javadi M. H. M. (2019). Application of the stimuli-organism-response framework to factors influencing social commerce intentions among social network users. Int. J. Business Inf. Syst. 30, 177–202. doi: 10.1504/IJBIS.2019.097534

[B8] Dezfoulian D. Nemati A. (2025). From playground to cultural ground: the youth-driven revival of traditional iranian games for intangible cultural heritage preservation. Museum Int. 77, 118–131. doi: 10.1080/13500775.2025.2540217

[B9] Doan T. Ong D. C. Wu Y. (2025). Emotion understanding as third-person appraisals: integrating appraisal theories with developmental theories of emotion. Psychol. Rev. 132:130. doi: 10.1037/rev0000507 39298223

[B10] Edson G. (2004). Heritage: pride or passion, product or service? Int. J. Heritage Stud. 10, 333–348. doi: 10.1080/1352725042000257366

[B11] Fadlah N. S. H. Shukor R. A. Isnin A. A. Azmin A. F. Jamal A. F. (2025). Personal visuals and belonging: exploring emotional ties in family history narratives. Quantum Journal of. Social Sci. Human. 6, 460–468. doi: 10.55197/qjssh.v6i3.559

[B12] Falk C. F. Vogel T. A. Hammami S. Miocevic M. (2024). Multilevel mediation analysis in R: a comparison of bootstrap and Bayesian approaches. Behav. Res. Methods 56, 750–764. doi: 10.3758/s13428-023-02079-4 36814007

[B13] Frautschi R. S. Dawlagala N. Klingemier E. W. England H. S. Sinclair N. R. Zins J. E. (2020). The use of eye tracking technology in aesthetic surgery: analyzing changes in facial attention following surgery. Aesthetic Surgery J. 40, 1269–1279. doi: 10.1093/asj/sjaa008 31956904

[B14] Giovannini E. C. Lo Turco M. Tomalini A. (2021). Digital practices to enhance intangible cultural heritage. Int. Arch. Photogrammetry Remote Sens. Spatial Inf. Sci. 46, 273–278. doi: 10.5194/isprs-archives-XLVI-M-1-2021-273-2021

[B15] Granic I. Morita H. Scholten H. (2020).Young people's digital interactions from a narrative identity perspective: implications for mental health and wellbeing. Psychol. Inq. 31, 258–270. doi: 10.1080/1047840X.2020.1820225

[B16] Henry J. Bai Y. Wlodkowic D. (2022). Digital video acquisition and optimization techniques for effective animal tracking in behavioral ecotoxicology. Environ. Toxicol. Chem. 41, 2342–2352. doi: 10.1002/etc.5434 35848752 PMC9826254

[B17] Ibrahim R. Yuningsih S. H. (2025). Dynamics of intergenerational transmission of traditional agricultural knowledge in the Dayak Kenyah community: challenges and opportunities for strengthening ethno-sciences in the modern era. Int. J. Ethno Sci. Educ. Res. 5, 101–106. doi: 10.46336/ijeer.v5i2.937

[B18] Krasich K. Huffman G. Faber M. Brockmole J. R. (2020). Where the eyes wander: the relationship between mind wandering and fixation allocation to visually salient and semantically informative static scene content. J. Vis. 20:10. doi: 10.1167/jov.20.9.10 32926071 PMC7490225

[B19] Lim S. H. Cho I. Y. (2022). A structural equation model of developing a partnership between pediatric nurses and parents of children with cancer in South Korea. J. Pediatr. Nurs. 63, e27–e35. doi: 10.1016/j.pedn.2021.10.021 34776314

[B20] Liu Q. I. Abd Latuff Z. Adnan W. H. (2024). A study on audience perception of short videos on intangible cultural heritage. Quantum J. Social Sci. Human. 5, 474–486. doi: 10.55197/qjssh.v5i6.533

[B21] Lou S. Adzharuddin N. A. Zainudin S. S. S. Omar S. Z. (2025). Discrete emotions shape gender role attitudes: exploring the impact of gender-stereotyped Douyin urban romantic short dramas on Chinese youth. Stud. Media Commun. 13, 282–298. doi: 10.11114/smc.v13i3.7663

[B22] Mehravipour Z. Mehravipour M. (2025). Eye-tracking as a diagnostic tool for dyslexia ADHD stroke and Alzheimer's using average fixation duration metrics. Int. J. 10, 59–66. doi: 10.11648/j.ijpbs.20251003.11

[B23] Mesana J. C. B. de Guzman A. B. Valencia C. Q. Basister J. P. C. (2024). Mapping online viewers' social and non-social emotions using the lens of watching UNESCO cultural heritage sites' travel vlogs. J. Heritage Tour. 19, 696–713. doi: 10.1080/1743873X.2024.2327433

[B24] Nasir I. M. Khan M. A. Yasmin M. Shah J. H. Gabryel M. Scherer R. . (2020). Pearson correlation-based feature selection for document classification using balanced training. Sensors 20:6793. doi: 10.3390/s20236793 33261136 PMC7730850

[B25] Negi S. Mitra R. (2020). Fixation duration and the learning process: an eye tracking study with subtitled videos. J. Eye Mov. Res. 13:40. doi: 10.16910/jemr.13.6.1 PMC8012014 33828811

[B26] Okopnyi P. Guribye F. Caruso V. Juhlin O. (2023). Automation and redistribution of work: the impact of social distancing on live TV production. Hum. Comput. Interact. 38, 1–24. doi: 10.1080/07370024.2021.1984917

[B27] Orduna-Hospital E. Navarro-Marqués A. López-de-la-Fuente C. Sanchez-Cano A. (2023). Eye-tracker study of the developmental eye movement test in young people without binocular dysfunctions. Life 13:773. doi: 10.3390/life13030773 36983928 PMC10058691

[B28] Rwezaula A. A. Chachage B. L. Tonya E. M. (2022). The moderation effects of demographic variables on trust of mobile phone banking services; a case study of smallholder farmers in Dodoma Region, Tanzania. South Asian J. Social Stud. Econ. 14, 57–71. doi: 10.9734/sajsse/2022/v14i430394

[B29] Shakya M. Vagnarelli G. (2024). Creating value from intangible cultural heritage-the role of innovation for sustainable tourism and regional rural development. Eur. J. Cult. Manage. Policy 14:12057. doi: 10.3389/ejcmp.2024.12057

[B30] Sheets P. Rowling C. M. Gilmore J. Melcher N. (2023). Us and them: the role of group identity in explaining cultural resonance and framing effects. Mass Commun. Soc. 26, 252–274. doi: 10.1080/15205436.2022.2026399

[B31] Wang X. Zhang T. Duan L. Liritzis I. Li J. (2024). Spatial distribution characteristics and influencing factors of intangible cultural heritage in the Yellow River Basin. J. Cult. Herit. 66, 254–264. doi: 10.1016/j.culher.2023.11.024

[B32] Zhang L. Cui H. (2022). Reliability of MUSE 2 and Tobii Pro Nano at capturing mobile application users' real-time cognitive workload changes. Front. Neurosci. 16:1011475. doi: 10.3389/fnins.2022.1011475 36518531 PMC9743809

